# Nano-Based Drug Delivery or Targeting to Eradicate Bacteria for Infection Mitigation: A Review of Recent Advances

**DOI:** 10.3389/fchem.2020.00286

**Published:** 2020-04-24

**Authors:** Yuan-Chieh Yeh, Tse-Hung Huang, Shih-Chun Yang, Chin-Chang Chen, Jia-You Fang

**Affiliations:** ^1^Department of Traditional Chinese Medicine, Chang Gung Memorial Hospital, Keelung City, Taiwan; ^2^Program in Molecular Medicine, School of Life Sciences, National Yang Ming University, Taipei, Taiwan; ^3^School of Traditional Chinese Medicine, Chang Gung University, Taoyuan City, Taiwan; ^4^Graduate Institute of Health Industry Technology, Chang Gung University of Science and Technology, Taoyuan City, Taiwan; ^5^School of Nursing, National Taipei University of Nursing and Health Sciences, Taipei, Taiwan; ^6^Department of Cosmetic Science, Providence University, Taichung City, Taiwan; ^7^Chinese Herbal Medicine Research Team, Healthy Aging Research Center, Chang Gung University, Taoyuan City, Taiwan; ^8^Pharmaceutics Laboratory, Graduate Institute of Natural Products, Chang Gung University, Taoyuan City, Taiwan; ^9^Research Center for Food and Cosmetic Safety and Research Center for Chinese Herbal Medicine, Chang Gung University of Science and Technology, Taoyuan City, Taiwan; ^10^Department of Anesthesiology, Chang Gung Memorial Hospital, Taoyuan City, Taiwan

**Keywords:** nanomedicine, antibiotic, bacteria, drug delivery, drug targeting

## Abstract

Pathogenic bacteria infection is a major public health problem due to the high morbidity and mortality rates, as well as the increased expenditure on patient management. Although there are several options for antimicrobial therapy, their efficacy is limited because of the occurrence of drug-resistant bacteria. Many conventional antibiotics have failed to show significant amelioration in overall survival of infectious patients. Nanomedicine for delivering antibiotics provides an opportunity to improve the efficiency of the antibacterial regimen. Nanosystems used for antibiotic delivery and targeting to infection sites render some benefits over conventional formulations, including increased solubility, enhanced stability, improved epithelium permeability and bioavailability, prolonged antibiotic half-life, tissue targeting, and minimal adverse effects. The nanocarriers' sophisticated material engineering tailors the controllable physicochemical properties of the nanoparticles for bacterial targeting through passive or active targeting. In this review, we highlight the recent progress on the development of antibacterial nanoparticles loaded with antibiotics. We systematically introduce the concepts and amelioration mechanisms of the nanomedical techniques for bacterial eradication. Passive targeting by modulating the nanoparticle structure and the physicochemical properties is an option for efficient drug delivery to the bacteria. In addition, active targeting, such as magnetic hyperthermia induced by iron oxide nanoparticles, is another efficient way to deliver the drugs to the targeted site. The nanoparticles are also designed to respond to the change in environment pH or enzymes to trigger the release of the antibiotics. This article offers an overview of the benefits of antibacterial nanosystems for treating infectious diseases.

## Introduction

Microorganisms including viruses, bacteria, fungi, and parasites can cause infectious diseases. Infection-related illness is a leading cause of death globally. According to the report from World Health Organization, three infectious diseases were ranked in the top 10 death causes in 2016: lower respiratory infection (fourth place), diarrhea disease (ninth place), and tuberculosis (tenth place). Among the infectious microorganisms, bacteria remain the leading cause of death in children, the elderly and immunodeficient patients (Zhang L. et al., 2019). Pathogenic bacteria represent a main public health problem because of the high morbidity and mortality, as well as the increased expenditure on patient management (Woodford and Livermore, 2009). The development of antibiotics since the 1940s proved important in eradicating bacteria, thereby saving millions of patients' lives. However, the conventional antibiotics used for anti-bacterial therapy exhibit some limitations in modern medicine, including the low bioavailability, minimal permeation to the infection nidus, and the rise of drug-resistant bacteria (superbugs) (Pizzolato-Cezar et al., 2019). The abuse and misuse of antibiotics has led to the emergence of antibiotic-resistant bacteria threatening human health. Antimicrobial resistance has become one of the leading causes of death worldwide in recent years. Novel approaches for either the enhancement of the therapeutic efficiency of existing antibiotics or the development of new antibiotics are necessary to resolve antibiotic resistance. Currently, several strategies, such as chemical modification of antibiotics, combinatorial therapy, photothermal agents, antimicrobial peptides, cationic polymers, and nanoparticles have been reported to be useful for conquering antibiotic resistance (Gebreyohannes et al., 2019).

In the past decade, a great advance in nanomedicine holds promise for bacterial infection treatment. The nanoparticles can act as antibacterial agents or the carriers for loading antibacterial drugs to promote the bioavailability and effectiveness of antibiotics (Baptista et al., 2018). Antibacterial nanoparticles without the need of drugs are developed using diverse materials, including metals, chitosan, and surfactants (Taylor and Webster, 2011). The net-positive charge of cationic compounds can bind to the negatively charged membrane surface of bacteria, while the amphiphilic structure of some nanoparticles prompts membrane damage (Chen et al., 2012). The encapsulation of antibiotic drugs into nanocarriers is another strategy to enhance bacteria eradication and bioavailability. The delivery of the drugs from nanosystems improves the efficacy, while reducing the possible toxicity in comparison to conventional therapy. Due to the high surface-to-volume ratio, the possibility of surface functionalization, and the capacity to load drug molecules, nanoparticles contribute to the efficient antibacterial activity with their high affinity to bacteria (Zazo et al., 2016). The nanoparticles can protect the drugs from enzymatic attack and sustain the drug release to increase the half-life and bioavailability. The nano-sized nature is beneficial to extravagate through the endothelium in the inflammatory site for efficient accumulation in infectious nidus (Walvekar et al., 2019). The nanomedical strategy to improve antibiotic delivery for bacterial killing indicates the reduction of side effects and drug resistance.

**Figure 1:**
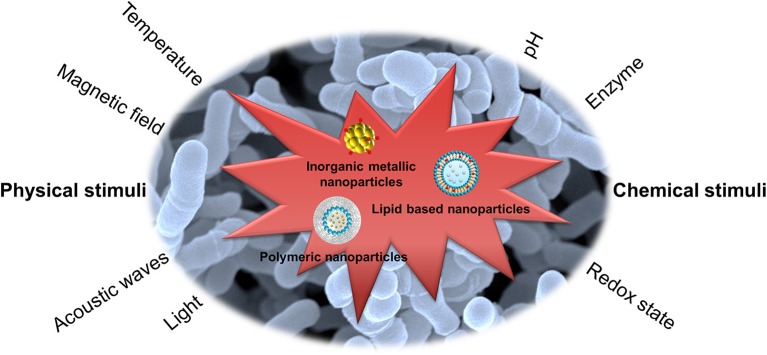
Stimuli-responsive nanoparticles for bacteria targeting via the recognition of bacterial microenvironment and the response in a dynamic process.

The active targeting of the nanoparticles to bacteria is an efficient management to increase the therapeutic index. The nanocarriers can be functionalized with ligands to the bacterial surface to enhance the targeting to specific pathogens. The design of stimuli-responsive nanosystems, as illustrated in Figure 1, is another concept for bacteria targeting through the recognition of bacterial microenvironment and the response in a dynamic process (Canaparo et al., 2019). The nanoparticles, after a suitable design, can respond to the internal stimuli such as varying pH, concentrations of specific enzymes, or chemicals, which are associated with pathological conditions of infection and inflammation (Lee et al., 2018). The antibiotic targeting to bacteria can be also achieved by nanoparticulate response to external physical stimuli, such as magnetic, thermal, light, and ultrasound effects. The application of stimuli to nanoparticulate drug delivery systems leads controlled drug delivery and fast response, addressing the pathological events. In addition, the reversibility to the initial state of the nanoparticles is possible to govern the antibacterial effect.

Besides the occurrence of drug-resistant bacteria, the infections associated with biofilm and intracellular residence are difficult to treat due to the inherent resistance to antimicrobial agents and immune cells (Yang et al., 2017). Biofilm is a sessile community of bacteria enclosed by the matrix of extracellular polymer substances (EPS) released by the microbes themselves. Biofilm reveals a rigid structure and high resistance that prevents the entrance of antimicrobial drugs (Mohammed et al., 2018). It is difficult to treat intracellular bacteria because these pathogens become recalcitrant via intracellular persistence in host cells (Cornejo et al., 2017). Some nanoparticles possess the ability to damage the biofilm for facile penetration into biofilm and subsequent bacteria eradication. The large uptake of nanoparticles into the host cells is possible through the functionalization of particulate surface for successful bacteria clearance. There are already 51 nanomedicines approved by the USFDA, including the nanoformulations for antibacterial aims (Bobo et al., 2016). In this review, we highlight recent advances in the application of nanomedicine for treating infection caused by bacteria. We mainly focus on the reports of nanoparticle development of antibacterial nanosystems during the past 5 years using different evaluation platforms, including *in vitro, ex vivo*, and *in vivo* examinations. The promising perspective in this emerging application is also discussed.

## The Bacteria Frequently Infecting Humans

Some bacteria strongly infect the human hosts to generate infectious diseases. The appearance of drug-resistant bacteria can worsen the condition of infection. Prior to the 1950s, antibiotics were extensively used in human medicine and animal agriculture due to their inexpensive cost and low side effects. However, the wide use of antibiotics over a period of several decades has resulted in the serious problem of drug resistance. Gram staining is a technique to differentiate two large groups of bacteria based on the different cell wall constituents (Thomson, 2016). The Gram stain procedure distinguishes between Gram-positive and Gram-negative groups by coloring these microbes violet or red. Gram-positive bacteria stain violet because of the presence of a thick layer of peptidoglycan in the cell walls retaining the crystal violet these cells are stained with. On the other hand, Gram-negative bacteria stain red attributing to a thinner peptidoglycan wall, which does not retain the crystal violet.

We introduce some bacteria frequently used as the cell models for testing the efficiency of nanoparticles on antibacterial assay. *Staphylococcus aureus* belongs to Gram-positive bacteria; it is the major consequence of bacterial infection in community settings and hospitals, eliciting significant morbidity and mortality (Tong et al., 2015). *S. aureus* has the capability to generate a diverse array of infection in different organs or tissues, including skin wound infection, folliculitis, pneumonia, endocarditis, and bacteremia (Price et al., 2017). Some of these diseases can threaten life. As an intracellular microbe, *S. aureus* is capable of invading macrophages, osteoblasts, and epithelial cells to evade immune surveillance (Fraunholz and Sinha, 2012). Multidrug-resistant *S. aureus*, especially methicillin-resistant *S. aureus* (MRSA), are a rising global health threat and economic burden. MRSA colonization is a predominant risk factor for adverse health outcome with 10–30% of carriers subsequently developing infectious disorders (Poovelikunnel et al., 2015). The resistance of MRSA to several antibiotics makes it necessary to use stronger antibiotics such as vancomycin. Unfortunately, many MRSA-infected patients do not respond favorably to vancomycin due to the recent development of vancomycin-resistant *S. aureus* (VRSA) and vancomycin-intermediate *S. aureus* (VISA) strains in clinics (Zhang et al., 2015). *Streptococcus pneumoniae* is a Gram-positive pathogen colonizing the upper respiratory tract. This strain infects the nasopharynx and spreads, especially in the form within the biofilm (Loughran et al., 2019). *S. pneumoniae* can be a leading cause of bacteremia, meningitis, otitis media, and community-acquired pneumonia. *S. pneumonia* produces >25,000 deaths of pneumonia patients >50 years each year in the US (Zivich et al., 2018). *Klebsiella pneumoniae* also causes pneumonia although it belongs to the Gram-negative strain. Besides pneumonia, this strain induces multiple infections, including bacteremia, meningitis, liver abscess, and urinary tract infection (Paczosa and Mecsas, 2016). Tigecycline is regarded as the effective and last-line antibiotic to treat *K. pneumoniae*. Nevertheless, the overuse of this antibiotic on *K. pneumoniae*-infected patients has led to the increased drug resistance and reduced sensitivity to tigecycline.

**Figure 2:**
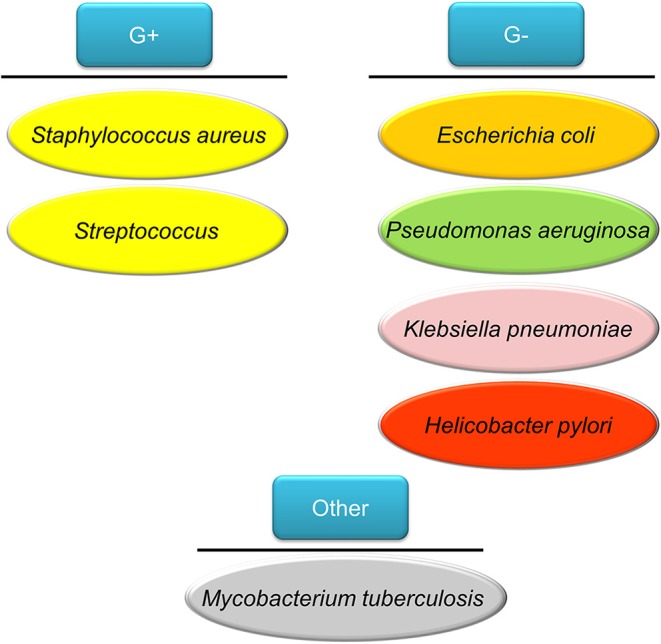
The bacteria commonly infect humans discussed in this review article as the model pathogens used for testing nanoparticle targeting.

People impaired with pulmonary mucociliary clearance are easily vulnerable to nosocomial infection, especially the Gram-negative *Pseudomonas aeruginosa*. The establishment of *P. aeruginosa* infection relates to the development of biofilm. The biofilm brings about the multidrug resistant *P. aeruginosa* strains (Amin and Ratjen, 2014). This pathogen is associated with cystic fibrosis and chronic obstructive pulmonary disease (COPD) (Hadinoto and Cheow, 2014). *Helicobacter pylori* is a Gram-negative pathogen affecting >50% of the global population (Bocian and Jagusztyn-Krynicka, 2012). *H. pylori* releases urease to hydrolyze urea to ammonia and bicarbonate for neutralizing acidity of stomach pH from 1–3 to 4.5–7, which is a favorable environment for *H. pylori* colonization (Ansari and Yamaoka, 2017). This pathogen plays an important role to elicit chronic gastritis, peptic ulcer, and gastric cancer. The emerging mutation of *H. pylori* has led to resistance to some antibiotics such as clarithromycin, resulting in the failure of therapy (Alba et al., 2017). The tuberculosis induced by *Mycobacterium tuberculosis* infection is one of the top ten causes of death worldwide, with more than 10 million people infected with tuberculosis each year. *M. tuberculosis* possesses a waxy coating on the cell surface because of the presence of mycolic acid. This coating makes the cells impervious to Gram staining. Thus, this pathogen can appear either Gram-positive or Gram-negative staining. Intracellular *M. tuberculosis* in host cells is able to bypass immune defense and modify its metabolic state. The emergence of drug-resistant *M. tuberculosis* has led to the failure of first line therapy and prolonged treatment duration (Seung et al., 2015). The above bacteria commonly infect individuals and the associated diseases are summarized in Figure 2.

## The Different Phases of Bacteria

**Figure 3:**
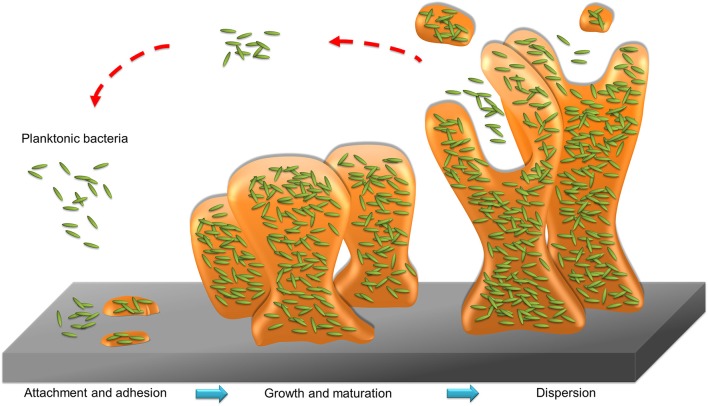
The formation process of biofilm.

Bacteria in the state of planktonic, biofilm, or intracellular form can infect humans. Planktonic bacteria are free-living bacteria. They live as floating microorganisms in their respective environments. The opposite mode of planktonic bacteria growth is the adherent or sessile type of growth. The bacteria can form biofilm or reside in the host cells. Biofilm and host cells can serve as shelters for bacteria, preventing the attack of antibiotics. Biofilm consists of microbes with altered phenotypes living in a self-organized community adhered to the surface or biomembrane. Bacteria in biofilm show different physiologies compared to the planktonic state, such as a diminished metabolic rate and increased communication between cells (Stewart and Franklin, 2008). The matrix of biofilm is mainly composed of polysaccharides, nucleic acids, lipids, and proteins, as shown in Figure 3. This matrix constitutes the protective microenvironment for bacterial colonization and fosters the formidable barrier to antimicrobial agents and the immune system (Di Martino, 2018). Biofilm offers a beneficial environment for gene transfer between the individual bacteria, spreading antibiotic resistance and making the bacteria more virulent (Lebeaux et al., 2014). The biofilm bacteria display a 10- to 1,000-fold higher resistance to antibiotic treatment than the planktonic form (Gebreyohannes et al., 2019). Infection associated with surgical devices and medical implants are always caused by the biofilm adherence. This virulent infection by biofilm evokes a high morbidity in hospitals. Surgical ares infection accounts for about 22% of hospital setting-acquired infection (Magill et al., 2014). The biofilm bacteria release toxins to cause complications like sepsis and hemorrhage shock. For instance, catheter-related sepsis costs an additional USD 57,090 per case (Nakamura et al., 2015). Antibiotic delivery by nanoparticles can be a promising approach to overcome the barrier function of biofilm. The use of fusogenic nanoparticles, nanoparticle targeting, and triggered drug release from nanocarriers are the strategies to maximize the exposure of the biofilm to drugs (Forier et al., 2014). Enzymes such as deoxyribonuclease (DNase) and protease are loaded into nanoparticles to hydrolyze the biofilm structure for enhanced penetration of antibacterial drugs or nanoparticles.

The transportation of bacteria into host cells is a process for inducing intracellular infection. The immune cells are the predominant cells for the intracellular invasion of the bacteria. For instance, macrophages are recruited to the infection site to play a key role in the defense against pathogens. The bacteria facilely bound to macrophage membrane and subsequently are internalized into phagosomes. Some microbes escape the macrophage-mediated disruption via a tumor necrosis factor (TNF) receptor-dependent pathway (Loeuillet et al., 2006), leading to the bacterial survival and enrichment. Another case is the bacteria infection into neutrophils. The bacteria possibly survive in neutrophils for a prolonged duration. The intracellular residence in the neutrophils protects the bacteria from lethal action by host immune system (Medina et al., 2003). Most of the antibacterial agents demonstrate limited activity against intracellular bacteria. The antibiotic concentrations of less than minimum inhibitory concentration (MIC) are usually found within the intracellular compartment, resulting in the development of drug resistance (Armstead and Li, 2011). Achieving efficient pathogen elimination requires maintaining a high antibiotic concentration in the host cells infected by bacteria. A number of nanoparticles, such as liposomes, polymeric nanoparticles, lipid-based nanoparticles, and silica nanocarriers, are produced for facile internalization into the host cells to eradicate intracellular microbes (Zhang L. et al., 2010). The intracellular nanoparticles can be designed through the increased affinity to host cells or the ligand conjugation on nanoparticulate surface for active targeting to cells.

## Nanoparticle Types Used For Antibacterial Application

**Figure 4:**
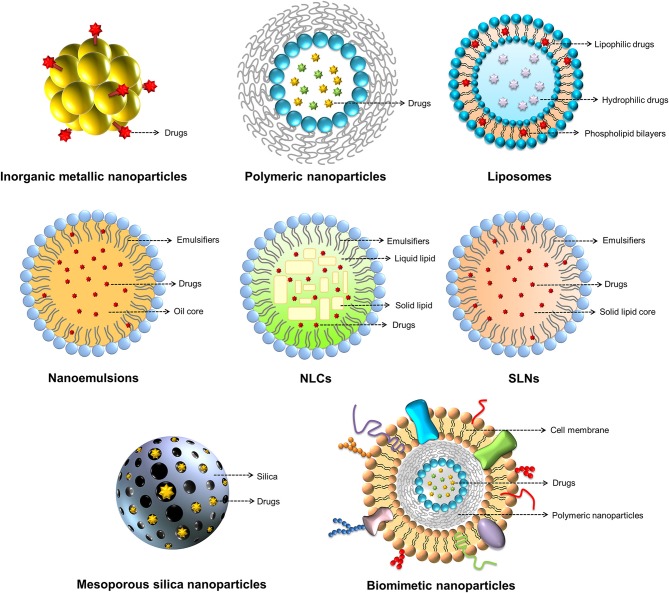
The nanoparticle classes applied for antimicrobial chemotherapy.

The nanoparticles can be made by a variety of materials to serve different purposes. There are different types of nanoformulations employed for antibacterial application. For drug delivery, the nanoparticulate core or shell can load several payload drugs. The shape, size, and surface charge of the nanoparticles can be finely tuned by modulation of material types, contents and preparation processes for optimizing drug release and organ/cell targeting (Palange et al., 2014). Inorganic metal, polymeric, lipid-based, micellar, silica, and cell membrane-coated nanoparticles are the commonly studied nanosystems for antibiotic drugs (Makowski et al., 2019). Figure 4 summarizes the nanoparticle classes applied to antimicrobial chemotherapy.

Metallic nanoparticles mainly made by Au, Ag, or Cu are found to present strong antimicrobial activity (Miller et al., 2015). However, the application may be hindered because of their potential toxicity to mammalian cells. Reforming of the metallic nanoparticles is needed to improve the biocompatibility. Compared to the other inorganic metal nanoparticles, Au nanoparticles are attracting great attention due to their acceptable biocompatibility, stable storage, and easy surface functionalization (Cabuzu et al., 2015). Due to the unique physical and chemical characteristics, Au nanoparticles have been extensively applied in drug delivery carrier, bioimaging, and anticarcinogenic therapy (Ramalingam, 2019). The adsorption of drug molecules on the Au particle surface allows the delivery of active ingredients to target sites. Some antimicrobial agents such as antibiotics, antibacterial peptides, and surfactants can be conjugated onto the nanoparticulate shell to initiate potential bactericidal activity (Yougbare et al., 2019). Ag nanoparticles themselves reveal a broad spectrum to eradicate bacteria, including some drug-resistant strains (Kasithevar et al., 2017). Nano-sized Ag shows greater biocidal effect than the bulk material (Choi and Hu, 2008). The mechanisms of killing bacteria by Ag nanoparticles are the disintegration of bacterial wall and the subsequent leakage of cytoplasmic contents and inactivation of proteins responsible for DNA and RNA replication. The superparamagnetic iron oxide nanoparticles (SPIONs) are widely investigated as powerful bactericidal agents due to their magnetic hyperthermia property (Javanbakht et al., 2016). Moreover, SPIONs are applicable as bacteria separation agents and bioimaging contrast agents for bacteria diagnosis (Sabale et al., 2017). SPIONs adsorb electromagnetic radiation and then convert the magnetic energy into heat under a magnetic field with high frequency and amplitude. The hyperthermia causes the increased bacterial membrane permeability to kill the targeted bacteria since most bacteria become vulnerable at the temperature of >45°C (Ibelli et al., 2018). In order to potentiate the antibacterial activity, SPIONs can be functionalized with antibodies, antimicrobial peptides, and aptamers for targeting specific bacteria (Chen et al., 2017). The magnetic nanoparticles made with iron oxide are also effective in deep penetration into biofilm by the triggering of a magnetic field (Park et al., 2011).

Natural or synthetic polymers can be utilized to fabricate nanoparticles for biomedical use. The antibiotics can either be covalently bound to a polymer backbone or physically incorporated into a polymer matrix. The biopolymers can form nanoparticles with high biocompatibility and biodegradability. They are classified into polysaccharides, nucleic acids, and peptides/proteins. Chitosan is one of the biopolymers with linear polysaccharide composed of randomly distributed β-(1 → 4)-linked D-glucosamine and *N*-acetyl-D-glucosamine. Chitosan itself can act as antibacterial and antibiofilm agents due to its polycationic nature's ability to disrupt bacterial membrane (Khan et al., 2020). Chitosan-based nanoparticles have been broadly used as drug delivery systems. The mucoadhesive character of chitosan nanoparticles contributes to the prolonged residence time in biomembranes, such as cornea, gastrointestinal epithelium, and buccal mucosa for sustained drug release (Quiñones et al., 2018). Alginate is another biopolymer commonly used to fabricate drug delivery nanocarriers. Contrary to chitosan, alginate is the anionic polysaccharide derived from the cell wall of algae. Alginate-based nanoparticles are reported to load antimicrobial agents for treating tuberculosis and fungal infection (Jana et al., 2016). Proteins are interesting ingredients for the preparation of nanoparticles because of the variety of molecular weights and easy chemical modification. Several proteins have been employed to develop nanodelivery systems, such as heat shock albumin, proteins, and ferritin (Tarhini et al., 2017).

Although the synthetic polymers meet the challenges in regard to their biocompatibility and biodegradability, recent studies prove that some synthetic polymers can be generally regarded as safe (GRAS) as recognized by the USFDA. An example is poly(lactide-*co*-glycolide) (PLGA). This polymer can be hydrolyzed to non-toxic oligomers or monomers of lactic acid and glycolic acid. PLGA nanoparticles are designed for drug delivery to aid therapeutic efficacy by drug protection, prolonged residence time, and nidus-targeting ability (Swider et al., 2018). According to the industrial consideration, there are many procedures for fabricating PLGA nanoparticles. Most of these techniques are easy to scale-up (Kim et al., 2019). The drug release and degradation rate can be tuned and controlled by changing the ratio of lactic acid and glycolic acid. Another case of synthetic polymers with acceptable biocompatibility is poly(malic acid) (PMLA), a biocompatible amphiphilic polymer based on polyesters. The features of PLMA are water soluble, biodegradable, and less toxic (Loyer and Cammas-Marion, 2014). The pendent carboxyl moieties in PLMA enable the introduction of various chemical modifications for nanoparticle development; these include antibodies, proteins, and specific drugs, including antibiotics (Chi et al., 2016).

Lipid-based nanoformulations, such as liposomes, nanoemulsions, and solid lipid nanoparticles (SLNs), are frequently applied for transporting antibacterial drugs. Lipid nanoparticles can facilely fuse with bacterial membrane, delivering antibiotics directly to bacteria (Furneri et al., 2017). Liposomes, as the carriers for drug delivery, can prolong circulation time and accelerate cellular uptake, thus countering therapeutic resistance; these nano-sized vesicles consist of membrane-like phospholipid bilayers in an aqueous solution (Fenske and Cullis, 2008). Liposomes have gained much attention because of their non-toxicity and structural similarity to cells. Liposomes can fuse with mammalian cells, tumor cells, and microbes, facilitating the transport of drugs across biomembranes (Fang et al., 2014). Lipid nanocarriers, such as solid lipid nanoparticles (SLNs), nanostructured lipid carriers (NLCs) and nanoemulsions, appear suitable as drug-carrier systems due to their very low cytotoxicity relative to polymeric nanoparticles (Wen et al., 2011). The predominant difference among SLNs, NLCs and nanoemulsions is the composition of the inner core. SLNs are particles made from crystalline solid lipids, whereas NLCs are composed of a solid lipid matrix with a certain content of a liquid lipid; they are a more advanced generation of SLNs. Nanoemulsions are nanocarriers with neat liquid oil in the inner phase. These lipid nanocarriers were introduced as antibacterial drug carriers for targeting bacteria and diminishing biofilm (Forier et al., 2014).

Because of their physicochemical stability, uniform porosity, great surface area, and biocompatibility, mesoporous silica nanoparticles (MSNs) are widely employed as drug delivery carriers, biosensors, catalysts, and adsorbents (Hao et al., 2017). MSNs with tunable particle size, pore volume, and morphology are promising carriers for drug delivery. The antibacterial agents inside the porous matrix are effectively shielded against enzymatic degradation (Bernardos et al., 2019). The surface chemistry of MSNs can be modified to facilitate the passage through biomembranes. The coating of natural cell membrane on nanoparticles has gained much attention recently. This strategy leverages native cell function for improving therapeutic effect. The biomimetic nanoparticles show therapeutic benefits, including prolonged nanoparticle circulation, cell-specific targeting, and immune system targeting (Gao and Zhang, 2015). The nanoparticles can be coated with the membranes of cancer cells, erythrocytes, neutrophils, macrophages, or platelets to show their capability to bind with the source cells (Vijayan et al., 2018). The platelet membrane-coated nanoparticles are able to mimic the platelet binding with bacteria for targeted antibiotic therapy (Kroll et al., 2017).

## The Interaction Between Nanoparticles and Bacterial Membrane

The first step of nanoparticles for killing bacteria is the interaction with bacterial surface. There are some reports describing the interaction between nanoparticles and bacterial wall/membrane, especially the metal-based nanoparticles. The bacterial surface is basically characterized by cell wall and cell membrane. The bacterial wall lies outside the membrane with a function to maintain the osmotic pressure of the cytoplasm and cellular morphology. Bacterial wall is composed of a homogeneous peptidoglycan layer consisting of sugars and amino acids. Gram-positive strain has one cytoplasmic membrane with multilayer of peptidoglycan polymer and a thicker wall (Fu et al., 2005). On the other hand, the Gram-negative strain wall is composed of two membranes, an outer membrane and a plasma membrane with a thin peptidoglycan layer. Nanoparticles and bacteria can interact intimately. The direct physical interaction of nanoparticles with bacteria is possible. This association is the nanoparticle anchoring onto bacterial wall or incorporation into the bacterial surface (Shrivastava et al., 2007). It is also possible that inorganic metal nanoparticles have the capability to induce irregular pit formation on the bacterial wall to facilitate ions penetrating into the cytoplasm (Pal et al., 2007). The nanoparticle interaction with cell wall can alter the membrane potential to enhance microbial membrane permeability (Vazquez-Muñoz et al., 2019). The dissipation of plasma membrane potential by the metallic nanoparticles leads to ATP depletion and outer membrane destabilization (Lok et al., 2006). Another case is that carbonaceous C_60_ fullerene nanoparticles can directly interact with bacteria to show cell membrane damage without the influence of energy metabolism (Tang et al., 2007).

The physicochemical features of the nanoparticles such as size, shape, and surface charge are vital to govern the bacterial interaction and antibacterial activity. The size has been shown to largely affect nanoparticle interaction with bacteria. In general, smaller-sized nanoparticles have the higher possibility to interact with bacteria and the following antibacterial potency. The smaller nanocomposites reveal higher surface area-to-mass ratio for increasing the adaption and binding to bacterial surface (Aruguete and Hochella, 2010). Also, the smaller size can facilely permeate into the bacterial membrane to manifest greater antibacterial activity (Zhang et al., 2007). The smaller nanoparticles have been shown to create or release more radicals, which are the important factors to eradicate pathogens (Applerot et al., 2012). In the case of metallic nanoparticles, the smaller-sized nanomaterials increase the dissolution rate of the ions from the particles for association with bacterial surface (Pareek et al., 2018). The increased ion dissolution results in the potentiation of bacterial killing. Nanoparticles may aggregate into larger clusters before and after attaching onto bacterial surface (Tamayo et al., 2014). The nanoparticle aggregates with larger size still can contact the bacterial surface but different manners as compared to the dispersed nanoparticles (Liu et al., 2009). Recent study (Kerisit and Liu, 2009) demonstrated that the aggregates reduced the reactivity with bacteria, resulting in the lower impact upon bacteria. Although it is generally recognized that the smaller-sized nanoparticles have higher ability to eradicate bacteria, however, it cannot be ignored that some studies (El Badawy et al., 2011; Sohm et al., 2015) claimed that the larger nanoparticles are more effective to kill bacteria. In addition to the size, other physicochemical properties, the bacteria model used, and experimental environment are factors influencing bacterial interaction and the following killing.

The most common shape observed for nanoparticles is spherical type. The other shapes including tube, rod, cube, sheet, plate, triangle, and pyramid are also reported (Slavin et al., 2017). The shape is expected to play a role on the interaction with bacteria. The rod or cube shape seems to be more effective to interact with bacteria than the other types. It can be due to the effect of crystal facet (Wang et al., 2014). The rod-shaped metal nanoparticles possess (111) and (100) facets, whereas the spherical nanoparticles have the (100) facets (Pal et al., 2007). The higher facets with greater atom density exhibit less energy to form oxygen vacancies, linking the greater antibacterial activity. Moreover, the rod or wire morphology of nanoparticles facilely penetrates into bacterial wall as compared to spherical type (Yang et al., 2009). The previous study (Talebian et al., 2013) also indicated a greater biocidal activity of flower-shaped nanoparticles against *S. aureus* than the spherical nanoparticles. Electrostatistical adsorption is an important interaction between nanoparticles and bacterial membrane. Positively charged nanoparticles display the strong affinity to negatively charged bacterial surface (Tang et al., 2007). Positively charged nanoparticles tightly adhere to the bacterial surface, followed by the fusion with cell wall, while no attachment was detected for the negatively charged nanoparticles in the case of Ag nanoparticles (Ivask et al., 2014). The cationic nanoparticles also alter the function of electron transport chain in microbes (Slavin et al., 2017). The positive charge in the nanoparticles has led to the damage on cell membrane, which is a mode of antibacterial action. The functionalization of nanoparticulate surface also causes a better interaction and bactericidal activity. For instance, the carboxylic acid-functionalized Au nanoparticles show a preferential attachment to the sub-polar region of bacterial membrane (Jahnke et al., 2016). However, some functionalizations of nanoparticles such as PEGylation and antibody conjugation may retard the attachment to bacteria because of the steric hindrance.

## The Possible Mechanisms of Antibacterial Nanoparticles

Some nanosystems represent antimicrobial activity without the inclusion of antibiotics. These include metal-based, surfactant-decorated, and chitosan nanoparticles. The bacterial killing mechanisms of these antibacterial nanoparticles include membrane disruption, reactive oxygen species (ROS) production, ATP depletion, and DNA synthesis inhibition (Slavin et al., 2017; Tamara et al., 2018). Nanoparticulate adsorption to cell surface causes the depolarization, which changes the typically negative charge of the wall to become more permeable. The bacterial wall would be blurry and degraded to produce the cytoplasm material leakage (Shmarakov et al., 2014). This phenomenon can be found for both Gram-positive and Gram-negative species. The metal ions or cationic surfactants released from the nanoparticles can directly interact with the bacterial membrane to generate membrane destabilization.

ROS production is recognized to represent an essential role of bacteria killing by antibacterial nanoparticles, particularly the metallic nanocomposites. The toxicity of ROS to the microbes is attributed to the high reactivity and oxidizing property (Zhang H. et al., 2010). Such ROS includes superoxide anion, hydrogen peroxide, and hydroxide. The toxicity of these species involves the destruction of DNA, lipids, and proteins after nanoparticle entrance into bacterial cytoplasm (Abo-Zeid and Williams, 2020). ROS is formed to suppress ATP generation and DNA replication (Ramalingam et al., 2016). For example, bacterial cells treated with Ag nanoparticles inactivate ribosomal subunit protein express and enzymes essential to ATP production (Yamanaka et al., 2005). The nanocomposites made by ZnO or TiO_2_ can generate oxidative stress on bacteria in the presence of UV radiation. ZnO nanoparticles can highly absorb UV light to induce phototoxic effect that produces superoxide anion and hydrogen peroxide (Sirelkhatim et al., 2015). These active species easily diffuses into the bacteria to kill microorganisms. TiO_2_ nanoparticles show antibacterial ability through the photocatalysed generation of radicals under UV irradiation (Brunet et al., 2009).

## Cell-Based Evaluation of Antibacterial Nanoparticles

### Antibacterial Nanoparticles Against Planktonic Bacteria

Planktonic bacteria are an original form of bacteria infecting humans; they also contaminate food and medical devices. There are different types of nanoparticles used to eradicate planktonic bacteria, including the multidrug-resistant strains. The methodology frequently used for evaluating the antimicrobial activity of the nanoparticles or compounds includes MIC, minimum bactericidal concentration (MBC), agar diffusion assay, live/dead assay, and colony-forming unit (CFU) quantification. Clinical and Laboratory Standard Institute (CLSI) provides the standard protocol to estimate MIC and agar diffusion analysis, which are the commonly used approaches for examining bacterial growth inhibition. However, the protocol is usually modified to change the treatment periods, bacterial counts, culture media, and temperature in different investigations for the optimization of different bacterial strains and antimicrobial nanoparticles/compounds. Pradeepa et al. (2016) prepared Au nanoparticles by using bacterial exopolysaccharide as the stabilizing agent for treating multidrug-resistant bacteria. The fluoroquinolone antibiotics were loaded into the Au nanoparticles. The nanosystems revealed reduced MIC and MBC against drug-resistant *S. aureus, K. pneumoniae*, and *E. coli* compared to the free drugs. In the inhibition zone determination, levofloxacin-, ceftriaxone-, and cefotaxime-loaded nanocarriers at 5 μg/ml exhibited the largest inhibition zones of 7.7, 10.7, and 11.6 mm against *S. aureus, K. pneumoniae*, and *E. coli*, respectively. The inhibition of complete bacteria growth could last up to 10 h for the nanoparticles. Antimicrobial peptides are found in nature. More than 2,000 antimicrobial peptides are demonstrated as effective antimicrobials against a broad spectrum of strains (Mikut et al., 2016). The conjugation of antimicrobial peptides in Au nanoparticles can ameliorate the low stability of these peptides. Casciaro et al. (2017) established the covalent conjugation of antimicrobial peptide esculentin-1a to Au nanoparticles via polyethylene glycol (PEG) linker. This nanoformulation increased the anti-*P. aeruginosa* activity of free esculentin-1a by 15-fold without being toxic to human keratinocytes. The nanoparticles could bear the proteolytic digestion for protecting the peptide.

Bajaj et al. (2017) reported the development of antimicrobial dipeptide-stabilized Au or Ag nanohybrids. The Ag nanoparticles containing dipeptides showed MIC of 0.37–0.93 μM against *S. aureus*, whereas the Ag nanoparticles or dipeptides alone only exhibited the MIC of 2.47 or 24.16–50.83 μM. A synergistic effect could be observed by combining dipeptides and Ag nanoparticles. The dipeptide-capped Au nanoparticles showed no bacterial inhibition. Ag nanoparticles with the size of <30 nm tend to aggregate, resulting in decreased bactericidal activity. Kooti et al. (2018) found that graphene oxide coating on Ag nanoparticles was effective in inhibiting aggregation. The further incorporation of CoFe_2_O_4_ led to the possibility of magnetic targeting of bacteria. By using ciprofloxacin as the incorporated antibiotic, this nanosystem can release this drug in a controlled manner. The inhibition zone for the ciprofloxacin-loaded nanocomposite increased more than 2-fold compared to that of nanocomposite without drug, and much greater than that of ciprofloxacin alone. The nanocomposite was more effective in eradicating Gram-negative bacteria with an inhibition zone of 34–39 mm than Gram-positive bacteria (30–32 mm). Zomorodian et al. (2018) attempted to encapsulate magnetic Fe_3_O_4_ by PEG for improving hydrophilicity and biocompatibility. Ag as the antimicrobial agent was coated onto the nanoparticulate shell. The inhibition zone of *S. aureus* for this nanocomposite was 30 mm, which was comparable to the positive control of tetracycline (33 mm). The viability of adipose-derived mesenchymal stem cells was nearly 100% after the intervention of nanocomposite at 8 μg/ml, indicating its safe use. The biodegradable polycarbonates were grafted onto superparamagnetic MnFe_2_O_4_ nanoparticles for bacterial targeting (Pu et al., 2016). Cationic polycarbonates provide a strong interaction with bacterial surface. Upon increasing the concentration from 15 to 120 μg/ml, the killing percentage of the magnetic nanoparticles against *E. coli* rose from 3 to 97%. The antibacterial potency of the polycarbonate-MnFe_2_O_4_ nanoparticles was increased 3-fold compared to the polymer alone. The magnetic field was applied to the nanoparticles to produce hyperthermia. This thermal effect, by heating to 52°C, resulted in a killing efficiency of 97% at 8 μg/ml.

Chitosan is commonly used to prepare antibacterial nanoparticles. It has dual roles as bactericidal agent and mucoadhesive material in nanoparticles. Chitosan nanoparticles were used to entrap daptomycin for *Staphylococcus* bacteria, including MRSA (Silva et al., 2015). The encapsulation percentage of daptomycin in nanocarriers was >80%. The complete daptomycin release from the nanocarriers was 4 h. Daptomycin-loaded nanocarriers showed anti-MRSA activity with MIC of 1–2 μg/ml. Ciprofloxacin was loaded into chitosan/heparin nanoparticles to target enteropathogenic bacteria (Kumar et al., 2016). The MIC against *E. coli* of free drug and ciprofloxacin-loaded nanocarriers was 0.25 and 0.125 μg/ml, respectively. The drug-loaded nanocarriers killed nearly 60% of bacteria within 30 min. The proposed bactericidal mechanism was the synergistic effect of the biopolymer and the drug on bacterial membrane disturbance. The combination of biopolymers and synthetic polymers can produce the ideal drug delivery system with suitable biocompatibility and mechanical property. Arif et al. (2018) developed pH-sensitive chitosan/PMLA nanoparticles loaded with amoxicillin for *H. pylori* eradication. This nanosystem was conjugated with cysteine for achieving mucoadhesive and anticoagulant properties. This thiolated nanoformulation could delay amoxicillin release in gastric acid and allow effective delivery to the *H. pylori* infection region. The bacterial growth inhibition after the treatment of amoxicillin-loaded pH-sensitive nanocarriers for 6 h was 49%, which was much higher than that of the nanocarriers without pH response (27%). Proteins represent a versatile biopolymer material for nanoparticle preparation. Steiert et al. (2018) prepared antibacterial protein-based nanoparticles consisting of PEGylated lysozyme. The native lysozyme was selected as the antibacterial agent due to its innate activity as natural antibiotic (Ibrahim et al., 2002). At a concentration of 0.32 μg/ml, the lysozyme-based nanoparticles revealed lower *M. luteus* growth compared to free lysozyme. An adhesive interaction of the nanoparticles with *M. luteus* surface contributed to a sustained release and prolonged attack against peptidoglycan layer.

PLGA is the synthetic GRAS polymer used alone or in combination with the other polymers to produce nanoparticles. The antibiotic rifampicin was loaded into PLGA-N-2-hydroxypropylmethacrylamide (HPMA) nanoparticles to evaluate the bactericidal activity against *M. tuberculosis* (Rani et al., 2018). The rifampicin release from nanocarriers could be maintained in a sustained fashion up to 70 h, while the free drug was completely released within 6 h. The bacterial inhibition by the nanoparticles was 4 times greater than the free control according to the MIC determination. Radovic-Moreno et al. (2012) developed vancomycin-loaded PLGA-poly(L-histidine)-PEG nanoparticles for treating *S. aureus* and *E. coli*. This nanosystem was designed to strongly bind to bacteria in acidity. This mechanism involved the pH response of nanoparticulate surface charge switching by selective protonation of imidazole moieties of poly(L-histidine) in acidic environment. The binding study demonstrated an increased binding of nanoparticles to bacteria by 4 to 6-fold through the pH change from pH 7.4–6.0. The nanosystem reduced the loss of antibacterial efficacy at low pH, with an elevation of MIC of 1.3-fold as compared to 2.0-fold for free vancomycin. The depletion of phosphate in the intestine is a major cue triggering bacterial virulence. It is important to maintain phosphate concentration to prevent virulence expression. Yin et al. (2017) synthesized phosphate- and polyphosphate-crosslinked PEG nanoparticles for sustained delivery of phosphate. The release of phosphate from PEG-based nanocarriers was sustained for 100 h. The nanoformulation was effective in suppressing pyoverdin and pyocyanin, the virulence biomarkers of *P. aeruginosa*.

The nanoparticulate surface functionalized by carbohydrate/glycan is reported to display antibacterial function via carbohydrate-mediated targeting by bacterial proteins (Xue et al., 2011). Eissa et al. (2016) constructed polymeric glycosylated nanoparticles to encapsulate ampicillin. Poly(N-butyl acrylate) was used as the nanoparticulate core. The ampicillin-loaded glycol-nanoparticles were found to elicit aggregation of *S. aureus* and *E. coli*, resulting in vital bacteria eradication by the antibiotic release in the proximity of the bacterial envelope. Curcumin as a natural compound is well-known for its antibacterial activity (Praditya et al., 2019). Shlar et al. (2017) developed polyquaternium-10-based nanoparticles for curcumin to display anti-*E. coli* activity. Polyquaternium-10 is a cellulose ether with cationic nature. The exposure of *E. coli* to the polyelectrolyte-coated nanoparticles inhibited bacterial proliferation with only minor viability reduction, suggesting bacteriostatic action. Both the bacterial membrane and ATP depolarization were the nanoparticle-related antibacterial mechanisms. Another polyelectrolyte nanosystem for bacteria eradication was the use of polyethyleneimine (PEI) and anionic enzyme-sensitive peptide for targeting *P. aeruginosa* (Insua et al., 2016). This enzyme-responsive nanosystem was selectively degraded in the presence of *P. aeruginosa* elastase, the virulence factor, while no degradation was detected in the presence of human neutrophil elastase. The nanoparticles exerted significant anti-*P. aeruginosa* activity without affecting other non-pathogenic strains.

Lipid-based nanoparticles offer high entrapment to lipophilic antibiotics. The polymers can be incorporated in lipid nanoparticles for developing lipid-polymer hybrid nanosystems. Dave et al. (2017) prepared norfloxacin-loaded hybrid nanoparticles by using soya lecithin and poly (lactic acid) (PLA) as the lipid and polymer components, respectively. The encapsulation efficiency of the antibiotic in the hybrid nanosystem was 72%. The drug release percentage reached 90% within 24 h. The norfloxacin-loaded nanocarriers retained antibacterial activity toward *S. aureus* and *P. aeruginosa* similar to free drug. The skin irritation of topically applied norfloxacin in rats was reduced after nanoparticulate incorporation. SLNs were fabricated to deliver antimicrobial oligonucleotide transcription factor decoys (TFD) (González-Paredes et al., 2019). The nanoparticles are helpful in protecting TDF from nuclease degradation and delivering TDF to target sites. SLNs were coated with either cationic bolaamphiphile 12-bis-tetrahydroacridinium or protamine to form the complexation with TFD. The cationic SLNs displayed extensive *E. coli* membrane binding and aggregation. A >2 log reduction in viable *E. coli* was found after nanoparticle treatment. The MIC was much less than the IC_50_ from cytotoxicity against Caco-2 cells, indicating the selectivity for bactericidal effect over mammalian toxicity. An acid-cleavable lipid was synthesized and used to develop pH-responsive SLNs for delivering vancomycin to acidic infection sites (Kalhapure et al., 2017). Vancomycin release from SLNs was faster at pH 6.5 than pH 7.4. *In vitro* antibacterial activity against MRSA showed that SLNs had enhanced activity at pH 6.5 than pH 7.4. The antimicrobial effect of NLCs loaded with nisin Z was evaluated by Lewies et al. (2017). Nisin Z is a cationic antimicrobial peptide produced by *Lactococcus lactis*. The incorporation of ethylenediaminetetraacetic acid (EDTA) in NLCs improved anti-*E. coli* activity due to EDTA's ability to destabilize the outer membrane to increase permeability. The antibacterial activity toward Gram-positive strains, such as *S. aureus* and *S. epidermidis*, also increased in the presence of EDTA. Another case of using lipid-based nanocarriers for protecting antimicrobial peptides is the lyotropic liquid crystalline nanostructure (Boge et al., 2016). The liquid crystallines consisting of cubic glycerol monooleate/water and hexagonal glycerol monooleate/oleic acid/water were examined as carriers for three antimicrobial peptides: AP114, DPK-060, and LL-37. The experimental data on MIC against MRSA demonstrated the superior activity of cubosomes over hexosomes, perhaps because the peptides tended to stay in the hexagonal structure with minimal release from the nanostructures.

Dendritic MSNs with center-radial pore architecture, large pore channel, and highly accessible pore volume have emerged as drug delivery system for large molecules such as proteins and DNA (Du and Qiao, 2015). Lysozyme, used as the antimicrobial enzyme to damage bacterial wall, was loaded into dendritic MSNs (Wang et al., 2016). The lysozyme-loaded nanocarriers had lower MIC against *E. coli* compared to free lysozyme (500 vs. 2,500 μg/ml). The MSNs released 67% lysozyme within 48 h. The total inhibition of *E. coli* growth by MSNs could be maintained throughout 5 d. The biomembrane-coated nanoparticles are applicable for on-demand antibiotic delivery. Li et al. (2014) prepared gelatin nanoparticles coated with red blood cell (RBC) membrane for delivering vancomycin. RBC membrane can reduce the clearance by the immune system and absorb bacterial exotoxin to relieve bacterial toxicity. The gelatin core was degraded by gelatinase, which is overexpressed in the infection microenvironment; the entrapped antibiotic was then released to kill the bacteria. Upon incubation with gelatinase-positive bacteria (*S. aureus* and *P. aeruginosa*), 62–92% vancomycin was released from the enzyme-responsive nanocarriers, whereas only 20% vancomycin was released in gelatinase-negative strains (*S. epidermidis* and *E. coli*). The MIC against *S. aureus* and *S. epidermidis* was 3 and 12 μg/ml, respectively. The antibacterial nanoparticles for eradication of planktonic bacteria evaluated via cell-based study are summarized in Table 1.

**Table 1:** The summary of antibacterial nanoparticles for eradication of planktonic bacteria evaluated via cell-based study.

**Nanoparticle type**	**Antibacterial agent**	**Average size**	**Bacteria strain**	**The feature of nanoparticles**	**References**
Au	Fluoroquinolones	20–30 nm	Drug-resistant *S. aureus, K. pneumoniae*, and *E. coli*	Exopolysaccharide as the stabilizing agent	Pradeepa et al., 2016
Au	Esculentin-1a	14 nm	*P. aeruginosa*	Increased esculentin-1a stability	Casciaro et al., 2017
Au or Ag	Dipeptides	12–15 nm	*S. aureus, E. coli*, and *S. typhimurium*	Both dipeptides and Ag as the antibacterial agents	Bajaj et al., 2017
Ag	Ciprofloxacin	15–16 nm	*S. aureus, B. subtilis, E. coli*, and *P. aeruginosa*	Combined with grapheme oxide and CoFe_2_O_4_	Kooti et al., 2018
Fe_3_O_4_-Ag	Ag	20–25 nm	*S. aureus* and *E. coli*	Increased hydrophilicity and biocompatibility by PEG	Zomorodian et al., 2018
MnFe_2_O_4_	Polycarbonates	17 nm	*S. aureus* and *E. coli*	Increased interaction with bacterial surface	Pu et al., 2016
Chitosan	Daptomycin	About 200 nm	*Staphylococcus* strains	Mucoadhesive property for ocular treatment	Silva et al., 2015
Chitosan/heparin	Ciprofloxacin	About 250 nm	*E. coli*	Synergistic effect of chitosan and antibiotic	Kumar et al., 2016
Chitosan/PMLA	Amoxicillin	186 nm	*H. pyroli*	pH-sensitive nanoparticles	Arif et al., 2018
PEGylated lysozyme	Native lysozyme	About 200 nm	*M. luteus*	Bioadhesive ability to bacteria	Steiert et al., 2018
PLGA-HPMA	Rifampicin	260 nm	*M. tuberculosis*	Sustained drug release	Rani et al., 2018
PLGA-poly(L- histidine)-PEG	Vancomycin	196 nm	*S. aureus* and *E. coli*	pH-sensitive nanoparticles	Radovic-Moreno et al., 2012
PEG	Phosphate and polyphosphate	About 180 nm	*P. aeruginosa*	Sustained phosphate release	Yin et al., 2017
Poly(N-butyl acrylate)	Ampicillin	302 nm	*S. aureus* and *E. coli*	Glycosylated nanoparticles for bacterial aggregation	Eissa et al., 2016
Polyquaternium- 10	Curcumin	146 nm	*E. coli*	A bacteriostatic action for the nanoparticles	Shlar et al., 2017
Poly(ethylene imine)	Poly(ethylene imine)	111 nm	*P. aeruginosa*	Targeting to *P. aeruginosa* elastase	Insua et al., 2016
Lipid-polymer hybrid	Norfloxacin	179–221 nm	*S. aureus* and *P. aeruginosa*	Less skin irritation	Dave et al., 2017
SLNs	Antimicrobial oligonucleotides	90–124 nm	*E. coli*	Protection oligonucleotides from enzymatic degradation	González-Paredes et al., 2019
SLNs	Vancomycin	133 nm	MRSA	pH-sensitive nanoparticles	Kalhapure et al., 2017
NLCs	Nisin Z	175–330 nm	*S. aureus, S. epidermidis*, and *E. coli*	Increased antibacterial activity by EDTA incorporation	Lewies et al., 2017
Lipid-based liquid crystals	Antimicrobial peptides	127–159 nm	*S. aureus*, MRSA, *P. aeruginosa*, and *E. coli*	Cubosomes showed superior antibacterial activity than hexosomes	Boge et al., 2016
Dendritic MSNs	Lysozyme	79–160 nm	*E. coli*	Prolonged bacteria growth inhibition	Wang et al., 2016
RBC membrane-coated nanoparticles	Vancomycin	97 nm	*S. aureus, S. epidermidis, P. aeruginosa*, and *E. coli*	Enzyme-sensitive nanoparticles	Li et al., 2014

*EDTA, ethylenediaminetetraacetic acid; HPMA, N-2-hydroxypropylmethacrylamide; MRSA, methicillin-resistant S. aureus; MSNs, mesoporous silica nanoparticles; NLCs, nanostructured lipid carriers; PEG, poly(ethylene glycol); PLGA, poly(lactide-co-glycolide); PMLA, poly(malic acid); RBC, red blood cell; SLNs, solid lipid nanoparticles*.

### Antibacterial Nanoparticles Against Biofilm Bacteria

Nanoparticles provide a unique approach to targeting bacterial biofilm. The nanoparticulate distribution through the biofilm layer is consistent with diffusive permeation. The small size of nanoparticles enables them to transport into the porous matrix (Botequim et al., 2012). It is generalized that nanoparticles diffuse via water-filled pores in the biofilm (Aljuffali et al., 2015). The biofilm is extremely non-wetting, limiting the penetration of some antimicrobial liquids (Sun et al., 2012). The low surface tension of nanoparticles assists the entrance into the non-wetting biofilm. Lambadi et al. (2015) conjugated polymyxin B, a cationic antimicrobial peptide, on Ag nanoparticle surface to assess the anti-biofilm activity against multidrug-resistant *P. aeruginosa*. The polymyxin B-capped Ag nanoparticles showed a 3-fold higher biofilm reduction than the neat Ag nanoparticles. In addition, the polymyxin B-capped nanoparticles removed 97% of the endotoxin of *P. aeruginosa*. Another inorganic metal nanosystem employed for biofilm disruption is Au (Giri et al., 2015). The Au nanoparticles were coated with thioalkyl tetra(ethyleneglycol)ated trimethylammonium and tetraethylene glycol to produce cationic and neutral particles, respectively. The positively charged nanoparticles displayed a significant reduction of *S. aureus* biofilm stained by crystal violet, whereas the neutral nanoparticles showed a lesser degree of diminishing biofilm. The cationic nanosystems are easily bound to biofilm polymers and negatively charged DNA. SPIONs can be targeted to the infection site under an external magnetic force to deeply penetrate into the biofilm (Subbiahdoss et al., 2012). Geilich et al. (2017) demonstrated biofilm growth inhibition by SPIONs decorated by PEG-*b*-PLA copolymer used to encapsulate methicillin. The application of SPIONs and magnetic field resulted in the deep penetration into *S. epidermidis* biofilm and selective bactericidal effect in the region of the applied magnetic field. The free antibiotic inhibited planktonic bacteria growth without the capability of entering the biofilm.

Chitosan with positive charge is applicable to produce anti-biofilm nanoparticles because of its affinity to polymers and DNA in biofilm. The *S. aureus* biofilm could be diminished by chitosan nanoparticles loaded with oxacillin and DNase I (Tan et al., 2018). DNase I is able to degrade extracellular DNA in biofilm matrix. The nanosystem with oxacillin and DNase I revealed greater biofilm eradication than did the nanoparticles without oxacillin or DNase I. The nanosystem could damage the biofilm via DNA degradation to kill *S. aureus* and reduce biofilm thickness. Repeated nanosystem treatment for 48 h induced a 98% biofilm reduction. Chitosan, PEG, and Fe_3_O_4_ were used to fabricate the biocompatible magnetic nanoparticles for biofilm eradication (Wang et al., 2018). Gentamicin was the selected antibiotic loaded in the magnetic nanocarriers. Under acidic condition, chitosan and PEG were protonated to facilitate the interaction with bacterial membrane. The magnetic field allowed deep penetration of the nanoparticles into mature *S. aureus* biofilm. The survival of bacteria in biofilm was about 80% and 5% after nanoparticle treatment without and with magnetic force, respectively.

With respect to PLGA nanoparticles, d'Angelo et al. (2015) designed PLGA/chitosan nanoparticles loaded with antimicrobial peptide colistin to eradicate *P. aeruginosa* biofilm. The nanocarriers were freeze-dried to produce the powder form for the future application in pulmonary inhalation. Free colistin at 7.5 and 15 μg/ml had a potent biofilm inhibition with 90% biomass reduction after 24-h treatment. This effect was diminished after 48 h and completely lost after 72 h. The nanocarriers could preserve anti-biofilm effect for 72 h. Baelo et al. (2015) developed ciprofloxacin-loaded PLGA nanoparticles functionalized by DNase I for eradicating *P. aeruginosa* biofilm. The nanoparticles showed a steady release of the antibiotic within 12 h. More than 80% of biofilm reduction was detected by nanoparticle treatment at the ciprofloxacin dose of 0.0156 μg/ml. The repeated application of the nanoparticles for 3 d resulted in biofilm reduction by 95%. Nguyen et al. (2016) determined anti-biofilm activity of poly(oligo(ethylene glycol) methyl ether methacrylate) (POEGMA) nanoparticles capable of storing nitric oxide and gentamicin. Nitric oxide is an agent used to provoke biofilm dispersal into antibiotic-susceptible planktonic form (Barraud et al., 2006). This nanosystem could simultaneously release nitric oxide and antibiotic to demonstrate a synergistic effect, eliminating biofilm and planktonic *P. aeruginosa* by 90 and 95%, respectively. The treatment of free nitric oxide or gentamicin only induced biofilm viability by <20%.

Liposomes are the lipid-based nanovesicles capable of loading antibiotics for biofilm eradication. Nisin was loaded into liposomes to clear the biofilm formed by *Streptococcus mutans* (Yamakami et al., 2013). The liposomes played a role in prolonging nisin release. There was 76% nisin released from liposomes within 6 h. The nisin concentration for efficacious glucan-biofilm inhibition was reduced 4-fold after liposomal encapsulation. This inhibition could be maintained during 6 h, while no inhibition was observed in the case of free nisin at this time. In another work, Ma and Wu (2016) prepared gentamicin-loaded liposomes to penetrate into alginate-based *Ralstonia insidiosa* biofilm in the presence of acoustic streaming created by pulsed ultrasound. The ultrasound pushed the liposomes into alginate-based biofilm. Afterward, the drug was released from liposomes disintegrated under pulsed ultrasound. This procedure reduced viable bacteria number in biofilm by 72%. Fang et al. (2019) evaluated the effect of cationic nanoemulsion droplet size on anti-biofilm activity against MRSA. The authors prepared three cetylpyridium chloride-coated nanoemulsions with average diameters of 55, 165, and 245 nm. The smaller droplets demonstrated potent anti-biofilm efficacy with a 10-fold reduction of bacteria survival compared with the non-treatment control. The loss of total DNA in bacteria was also increased following the decrease of droplet size. The smaller nanoparticles may be facilely delivered into the pores of the biomass to kill bacteria. Soyaethyl morpholinium ethosulfate (SME) is a cationic surfactant showing antibacterial activity through bacterial membrane lysis. Lin et al. (2017) compared the anti-biofilm activity of SME coated on nanoemulsions and liposomes. The data on MIC/MBC demonstrated superior antimicrobial effect of nanoemulsions compared to liposomes against MRSA and *S. epidermidis*. Nanoemulsions decreased MRSA biofilm thickness 2.4-fold, a result which was higher than liposomes (1.6-fold). The high surface charge, low lipophilicity, and wetting character of nanoemulsions contributed to the greater biofilm suppression as compared to liposomes. The antibacterial nanoparticles for eradication of biofilm bacteria are summarized in Table 2.

**Table 2:** The summary of antibacterial nanoparticles for eradication of biofilm and intracellular bacteria evaluated via cell-based study.

**Nanoparticle type**	**Antibacterial agent**	**Average size**	**Bacteria strain**	**The state of bacteria**	**References**
Ag	Polymyxin B	About 130 nm	*P. aeruginosa*	Biofilm	Lambadi et al., 2015
Au	Au	10-12 nm	*S. aureus*	Biofilm	Giri et al., 2015
SPIONs	Methicillin	About 50 nm	*S. epidermidis*	Biofilm	Geilich et al., 2017
Chitosan	Oxacillin	167 nm	*S. aureus*	Biofilm	Tan et al., 2018
Chitosan/PEG/ Fe_3_O_4_	Gentamicin	About 200 nm	*S. aureus*	Biofilm	Wang et al., 2018
PLGA/chitosan	Colistin	267 nm	*P. aeruginosa*	Biofilm	d'Angelo et al., 2015
PLGA	Ciprofloxacin	252 nm	*P. aeruginosa*	Biofilm	Baelo et al., 2015
POEGMA	Gentamicin	15 nm	*P. aeruginosa*	Biofilm	Nguyen et al., 2016
Liposomes	Nisin	Unknown	*Streptococcus mutans*	Biofilm	Yamakami et al., 2013
Liposomes	Gentamicin	182 nm	*Ralstonia insidiosa*	Biofilm	Ma and Wu, 2016
Nanoemulsions	Cetylpyridinium chloride	55, 165, and 245 nm	MRSA	Biofilm	Fang et al., 2019
Nanoemulsions and liposomes	Soyaethyl morpholinium ethosulfate	214 and 75 nm	MRSA	Biofilm	Lin et al., 2017
Chitin	Rifampicin	350 nm	*S. aureus, E. coli*, and *K. pneumoniae*	Intra-neutrophil infection	Smitha et al., 2015
Curdlan	Rifampicin and levofloxacin	619 nm	*Mycobacterium smegmatis*	Intra-macrophage infection	Yunus Basha et al., 2019

*MRSA, methicillin-resistant S. aureus; PEG, poly(ethylene glycol); PLGA, poly(lactide-co-glycolide); POEGMA, poly(oligo(ethylene glycol) methyl ether methacrylate); SPIONs, superparamagnetic iron oxide nanoparticles*.

### Antibacterial Nanoparticles Against Intracellular Bacteria

Two biopolymer nanoparticles were used for treating intracellular bacteria in recent years. Smitha et al. (2015) developed chitin nanoparticles for delivering rifampicin into neutrophils for killing intracellular pathogens. Rifampicin showed a sustained release from the nanoparticles till 72 h. The intracellular rifampicin concentration in neutrophils was enhanced 6- to 7-fold after loading in nanoformulation. The MIC of the nanoparticles to inhibit *E. coli* and *K. pneumoniae* was 20 and 35 μg/ml, respectively. This level was lower than that of free drug (35 and 40 μg/ml), perhaps due to the strong binding of the nanoparticles with bacterial surface. Macrophages are the immune cells serving as a host for *M. tuberculosis*. Rifampicin and levofloxacin were complexed with cyclodextrin and conjugated to curdlan-based nanoparticles for intra-macrophage *M. tuberculosis* eradication (Yunus Basha et al., 2019). Curdlan is a linear glucan derived from *Agrobacterium* and *Rhizobium*. It is recognized by dectin-1 receptor expression in macrophages (Ganbold and Baigude, 2018). The nanoparticle internalization by macrophages was 1.8-fold higher than that by fibroblast cells, indicating the usefulness of curdlan in nanoparticles for macrophage uptake. The nanocarriers could kill >95% of intra-macrophage bacteria within 4 h, whereas only 53% killing was detected in the group of free rifampicin. The antibacterial nanoparticles for treating intracellular bacteria are summarized in Table 2.

## Animal-Based Evaluation of Antibacterial Nanoparticles

### Skin and Subcutaneous Region Infection

The bacteria facilely locate in the skin, such as lesions of atopic dermatitis and chronic wounds, playing a critical role in infection-induced inflammation and cutaneous disease progression (Shi et al., 2018). The ideal infection model for assessing the antimicrobial activity of nanomedicine in animal-based studies is the skin and subcutaneous region infection, as one can observe the performance of bacterial infection due to the visible skin appearance. This infection model is also easy and simple to establish by topical bacteria administration or subcutaneous bacteria injection. Various administration routes of nanoparticle delivery can be used to treat cutaneous and subcutaneous infection, including topical, subcutaneous, and intravenous applications. Chitosan and 2-mercapto-1-methylimidazole (MMT) were coated on the surface of Au nanoparticles allowing multivalent interaction with bacterial membrane (Lu et al., 2018). The nanoparticles were crosslinked with gelatin to form wound dressing for treating skin infection. The MRSA-infected wound in rabbit back was topically administered by the nanoparticles. The regenerated skin after treatment of nanocomposite showed an appearance like normal skin, with 92% wound closure, whereas the wound treated by gauze only revealed 67% closure after 16 d. Liu et al. (2018) developed chemo-photothermal therapy against subcutaneous infection based on polydopamine-coated Au nanorods. Polydopamine coating achieved efficient antibacterial Ag loading in the nanorods. This platform became positively charged in the acidic environment of the bacteria-formed abscess, allowing their accumulation in the infection site as revealed by florescence imaging. The loaded Ag was released in a pH-sensitive manner. This nanosystem was intravenously injected into the mice to treat subcutaneous abscess under near infrared (NIR) irradiation. The hyperthermal effect of NIR led to more Ag release and MRSA killing for abscess ablation and wound healing acceleration.

Allicin is a natural antibacterial compound abundant in garlic (Wallock-Richards et al., 2014). Sharifi-Rad et al. (2014) combined allicin and antibacterial Ag nanoparticles for the management of skin infection in mice produced by MRSA. Topical ointment with allicin and Ag nanoparticles showed a synergistic effect in inhibiting MRSA infection in skin. The bacterial count for control, allicin, Ag, and the combination was 3.8 × 10^10^, 4.3 × 10^6^, 8.0 × 10^7^, and 0 CFU/ml, respectively. A photothermal nanocomposite of hyaluronic acid (HA)-templated Ag nanoparticles integrated with graphene oxide was prepared to treat *S. aureus* infection in skin (Ran et al., 2017). HA was degraded by hyaluronidase secreted from bacteria to trigger Ag release. NIR illumination on the nanoparticles containing graphene oxide locally raised the hyperthermia to eradicate the bacteria. In the *in vivo* skin wound infection study, the bacterial count of the combined nanoparticles and NIR was 2 orders less as compared to the control and NIR alone. Bacterial contamination in the central venous catheter (CVC) can result in bacterial consortium and inflammation. Ribeiro et al. (2018) immobilized SPIONs functionalized with antimicrobial peptide clavanin A on CVC for use as an antibacterial prophylactic. The skin infection in mice was induced by introducing CVC (40 mm in length) containing 20 μl of 1 × 10^9^ CFU/ml *K. pneumoniae*. After irradiation of diode laser (808 nm) on CVC for 5 min, the viability of the attached bacteria was reduced by 88%. The antimicrobial action could be maintained for 7 d. The inflammation was also reduced based on the determination of cytokines. The photothermal therapy was also established by using acetylcysteine-coated Prussian blue nanoparticles (Cai et al., 2019). Acetylcysteine is an antimicrobial agent with mucolytic activity. Prussian blue nanoparticles have been proven as a photothermal agent triggered by NIR (Szaciłowski et al., 2006). The acetylcysteine-coated nanoparticles were prepared based on the co-precipitation of K_4_Fe(CN)_6_ and FeCl_3_. The irradiation of NIR (980 nm) on nanoparticles at 50 μg/ml eliminated *S. aureus* and *E. coli* by 74 and 75%, respectively. The NIR exposure after subcutaneous injection of the nanocomposite generated local heat to eradicate *S. aureus* in subcutaneous abscess.

With respect to polymeric nanocarriers, poly(ε-caprolactone) (PCL) was used to incorporate carvacrol and then mixed with hydrogel for topical delivery (Mir et al., 2019). Carvacrol is a monoterpene showing bactericidal effect on a broad range of microbes (Nostro and Papalia, 2012). The nanoparticles were enzyme-sensitive to produce carvacrol release in the presence of bacterial lipase. The dermatokinetic study demonstrated an enhanced carvacrol deposition in epidermis from 0.04 to 0.96% of the applied dose after nanoparticle inclusion. The carvacrol-loaded nanoparticles in hydrogel showed MRSA burden reduction by 99.97% in the burn wound of pig skin. About 25% of bacteria residing in skin accumulated in hair follicles (Lange-Asschenfeldt et al., 2011). The bacteria deposited in hair follicles are usually difficult to eliminate. Hsu et al. (2017) developed chloramphenicol-loaded lipid-based nanocarriers for follicular delivery to eradicate MRSA. Dimyristoylphosphatidylcholine (DMPC) or deoxycholic acid (DA) was incorporated into liposomes for producing the malleable vesicles with the aim of easy extrusion into follicles. The flexible liposomes with DMPC and DA elevated intrafollicular uptake of the drug by 1.5- and 2-fold, respectively. *In vivo* topical application demonstrated found no skin irritation after the administration of liposomes for 7 consecutive d. Another case of lipid-based nanoparticles is the simultaneous incorporation of SME and oxacillin in NLCs for synergistic bactericidal effect against MRSA (Alalaiwe et al., 2018). The cationic NLCs could disorganize MRSA membrane to leak the proteins. This disintegration of membrane also promoted oxacillin entrance into the cytoplasm. Topical delivery of NLCs to MRSA abscess in mouse skin reduced MRSA load by 4 logs. The skin architecture and barrier function were also recovered by NLCs.

Yang et al. (2018) developed gentamicin-loaded MSNs coated with lipid bilayer surface. The bilayer shell of MSNs was decorated with *S. aureus*-targeting peptide ubiquicidin. The lipid bilayer could be degraded by bacterial toxins to rapidly release gentamicin. The fast antibiotic release was confirmed in the presence of planktonic and intra-macrophage *S. aureus*. The intracellular *S. aureus* was subcutaneously injected into mice. The mice were treated with nanocomposite via intravenous administration after 2 d. The bacterial load in the infected area was 2.3 × 10^7^ and 8.4 × 10^6^ CFU/ml upon the injection of PBS and free drug, respectively. The nanoparticles reduced bacterial burden to 1.5 × 10^4^ CFU/ml. Micelles are the supramolecular assembly of surfactants. The antibacterial SME was the cationic surfactant which could form micelles in a nanoscale size (Yang et al., 2016). In the mouse model of subcutaneous MRSA abscess, topically applied SME micelles demonstrated a reduced bacterial burden, compared to vehicle control, by 1.6 × 10^4^-fold. A negligible cutaneous irritation was found after micelle intervention on healthy mouse skin, suggesting safe application for anti-MRSA therapy. The antibacterial nanoparticles for treating topical infection in skin tissue are listed in Table 3.

**Table 3:** The summary of antibacterial nanoparticles for treating bacterial infection of skin and subcutaneous region evaluated via animal-based study.

**Nanoparticle type**	**Antibacterial agent**	**Average size**	**Bacteria strain**	**Animal model**	**References**
Au	Chitosan	8–13 nm	MRSA	Open wound infection in rabbits	Lu et al., 2018
Au nanorods	Ag	Length 68 nm; diameter 21 nm	MRSA	Subcutaneous abscess in mice	Liu et al., 2018
Ag	Allicin and Ag	10–30 nm	MRSA	Open wound infection in mice	Sharifi-Rad et al., 2014
Ag	Ag	20 nm	*S. aureus*	Open wound infection in mice	Ran et al., 2017
SPIONs	Clavanin A	10 nm	*K. pneumoniae*	Bacteria-containing CVC introduction in mice	Ribeiro et al., 2018
SPIONs	Acetylcysteine	95 nm	*S. aureus*	Subcutaneous abscess in mice	Cai et al., 2019
PCL	Carvacrol	164–233 nm	MRSA	Burn wound infection in pig skin	Mir et al., 2019
Liposomes	Chloramphenicol	132–239 nm	MRSA	Skin irritation test in nude mice	Hsu et al., 2017
NLCs	SME and oxacillin	177 nm	MRSA	Subcutaneous abscess in mice	Alalaiwe et al., 2018
MSNs	Gentamicin	95 nm	*S. aureus*	Subcutaneous abscess in mice	Yang et al., 2018
Micelles	SME	178 nm	MRSA	Subcutaneous abscess in mice	Yang et al., 2016

*CVC, central venous catheter; MRSA, methicillin-resistant S. aureus; MSNs, mesoporous silica nanoparticles; NLCs, nanostructured lipid carriers; PCL, poly(ε-caprolactone); SME, soyaethyl morpholinium ethosulfate; SPIONs, superparamagnetic iron oxide nanoparticles*.

### Pulmonary Infection

Bacteria can largely invade the human body via the respiratory tract to induce lung-related diseases such as pneumonia, tuberculosis, and cystic fibrosis. Some efforts are conducted to treat lung infection in animals by intravenous or intratracheal administration of nanoformulations. Kang et al. (2019) used tigecycline as the model antibiotic to be encapsulated in intercellular adhesion molecule (ICAM)1-conjugated β-Ga_2_O_3_:Cr^3+^ nanoparticles. ICAM1 is highly expressed in endothelial cells of inflammation sites. β-Ga_2_O_3_:Cr^3+^ is a semiconductor material with luminescent property for bioimaging (Wang et al., 2015). Tigecycline*-resistant K. pneumoniae* (TRKP) was administered into the lung via intratracheal route to establish the TRKP-infected pneumonia mice. Only the intravenous nanoparticle-treated mice demonstrated 100% survival against pulmonary infection after 12 d. The survival rate of the mice treated by free drug at 45 mg/kg was 83%, which was still lower than those treated by nanocarriers at 15 mg/kg. The *in vivo* biodistribution showed an increased fluorescence intensity of the nanoparticle-treated lung from 5 to 24 h post-injection, suggesting increased nanoparticle accumulation in the infected area through targeted delivery.

Some polymer-based nanocarriers are fabricated with the aim of treating *P. aeruginosa*-induced pulmonary infection. The inhaled tobramycin represents limited capability to penetrate DNA-rich lung mucus (Kłodzinska et al., 2016). Deacon et al. (2015) synthesized tobramycin-loaded chitosan/alginate nanoparticles functionalized with DNase for inhibiting mucus viscoelasticity by DNA cleavage. The treatment of the biopolymer nanoparticles prior to lung infection with *P. aeruginosa* offered longer protection, doubling the survival rate from 40% with free antibiotic to 80%. The nanoparticles containing DNase improved nanoparticle penetration in the sputum of cystic fibrosis patients. Casciaro et al. (2019) investigated lung infection treated by intratracheal PLGA nanoparticles containing antimicrobial peptide esculentin-1a. The nanoparticles were coated with poly(vinyl alcohol) (PVA) as the stabilizing agent. The neutral hydrophilic nanoparticle surface was favorable to permeate through pulmonary mucus. In the mouse model of lung infection induced by *P. aeruginosa*, the esculentin-1a-loaded nanocarriers reduced CFU by 3 logs as compared to PBS-treated group. This anti-*P. aeruginosa* activity was 17-fold stronger than that of free esculentin-1a. Amphiphilic PEG-*co*-PCL copolymer was conjugated with vancomycin as the targeting ligand via pH-cleavable hydrazone bonds to obtain micelle nanocarriers (Chen et al., 2018). Ciprofloxacin was loaded in the nanocomposite for on-demand release. The opening of the vancomycin shell of the nanocomposite under acidic environment could interfere with the hydrophilic/lipophilic balance, leading to micelle size enlargement, facilitating the degradation of PCL by lipase overexpressed in the infection site and ciprofloxacin release for *P. aeruginosa* destruction. The micelle treatment in *P. aeruginosa*-infected mice reduced the bacterial load and alveolar injury in the lung compared to free ciprofloxacin.

To deliver moxifloxacin to the infected lung tissue and enable sustained drug release, a ROS-responsive 4-(hydroxymethyl) phenylboronic acid pinacol ester-modified α-cyclodextrin was coated with phospholipids to form a lipid-coated nanoparticles for lung infection management (Wang et al., 2019). The coating with 1,2-distearoyl-sn-glycero-3-phosphoethanolamine (DSPE)-PEG-folic acid on nanocarriers helped to promote sputum penetration and targeting to the macrophages with overexpressed ROS in the inflammatory region. The nanosystem was intravenously injected into the mice with *P. aeruginosa* infection in lung. The administration of moxifloxacin could increase the survival rate from 20 to 40% after nanoparticulate encapsulation. Almost no pathogen colony was detectable in the lung after nanocomposite treatment. Another lipid-based nanoformulation investigated for ameliorating infectious pneumonia was PEGylated phosphatidylcholine-rich nanovesicles (Hsu et al., 2018). The nanovesicles were loaded with ciprofloxacin for lung targeting to pulmonary surfactants. This could lead to the eradication of intracellular MRSA. The *in vivo* biodistribution result showed a 3.2-fold increase of pulmonary ciprofloxacin accumulation after intravenous injection of the lipid nanovesicles. The pulmonary MRSA burden was restrained from 4.9 × 10^8^ to 1.2 × 10^8^ and 6.3 × 10^7^ CFU by treatment by control drug and the nanovesicles, respectively.

NZX is an antimicrobial peptide capable of suppressing the growth of drug-resistant *M. tuberculosis*. Tenland et al. (2019) aimed to entrap NZX into MSNs for treating tuberculosis because of the large uptake of MSNs by macrophages. The nanoparticles presented increased intra-macrophage bacteria killing compared to free NZX. In the murine tuberculosis model, the CFU of *M. tuberculosis* in the lung was decreased by 84% and 88% after intratracheal application of free peptide and NZX-containing MSNs, respectively. MSNs were also used for active targeting to treat pulmonary infection (Hussain et al., 2018). The vancomycin-loaded nanoparticles were conjugated with cyclic 9-amino-acid peptide CARGGLKSC (CARG), which could recognize *S. aureus*. CARG specifically bound to *S. aureus* but not *P. aeruginosa in vitro*. The intravenous CARG-conjugated nanoparticles exhibited 8-fold greater deposition in the lung compared to non-targeted nanoparticles. Intratracheal *S. aureus* instillation into the mouse lung led to 67% mortality after 24 h. This survival rate was increased to 100% by CARG-conjugated MSNs. All the mice treated with MSNs survived after 20 d. The antibacterial nanoparticles for treating pulmonary infection *in vivo* are listed in Table 4.

**Table 4:** The summary of antibacterial nanoparticles for treating bacterial infection of lung evaluated via animal-based study.

**Nanoparticle type**	**Antibacterial agent**	**Average size**	**Bacteria strain**	**Animal model**	**References**
β-Ga_2_O_3_:Cr^3+^	Tigecycline	10–20 nm	TRKP	TRKP-induced pneumonia	Kang et al., 2019
Chitosan/alginate	Tobramycin	505–538 nm	*P. aeruginosa*	*P. aeruginosa*-induced infection	Deacon et al., 2015
PLGA	Esculentin-1a	About 250 nm	*P. aeruginosa*	*P. aeruginosa*-induced infection	Casciaro et al., 2019
PEG-*co*-PCL	Ciprofloxacin	77 nm	*P. aeruginosa*	*P. aeruginosa*-induced infection	Chen et al., 2018
PSPE-PEG	Moxifloxacin	254 nm	*P. aeruginosa*	*P. aeruginosa*-induced infection	Wang et al., 2019
Liposomes	Ciprofloxacin	114 nm	MRSA	MRSA-induced pneumonia	Hsu et al., 2018
MSNs	NZX	About 200 nm	*M. tuberculosis*	*M. tuberculosis*-induced tuberculosis	Tenland et al., 2019
MSNs	Vancomycin	About 180 nm	*S. aureus*	*S. aureus*-induced infection	Hussain et al., 2018

*DSPE, 1,2-distearoyl-sn-glycero-3-phosphoethanolamine; MSNs, mesoporous silica nanoparticles; PCL, poly(ε-caprolactone); PEG, poly(ethylene glycol); PLGA, poly(lactide-co-glycolide); TRKP, tigecycline-resistant K. pneumonia*.

### Gastrointestinal (GI) Infection

Antimicrobial nanoparticles can be administered by oral route to treat GI tract infection. The nanocarriers protect the antibiotics from degradation in GI fluids. Some nanoparticles have the bioadhesive feature to prolong the retention in GI tract for enhanced oral bioavailability. Oral MSNs are ideal candidates to protect the drugs from enzymolysis in GI. Zhao et al. (2019) prepared antimicrobial peptide defensin-loaded MSNs for targeting the intestine. Defensin is easily degraded in the stomach. In order to achieve the intention of intestinal targeting, succinylated casein, that can be degraded by intestinal protease, was coated onto the surface of MSNs. Casein decoration decreased defensin release in acidic environment, whereas a controlled release fashion was found in the presence of trypsin. Multidrug-resistant *E. coli* was administered by oral gavage to evoke intestinal infection. The nanoparticles were orally administered every day for 5 d. The casein-coated nanomedicine significantly lowered bacteria colonization compared with free ciprofloxacin as the positive control. The proinflammatory mediator TNF-α in intestine was decreased by 1.5- and 2.2-fold after casein-coated nanoparticle application, compared to non-coated MSNs and free peptide, respectively.

Montmorillonite is a smectic clay material possessing the ability to attach bacterial EPS and mucoadhesive property (Calabrese et al., 2013). A metronidazole-loaded nanocomposite made with montmorillonite and cationic PEI was designed to treat *H. pylori* infection in GI (Ping et al., 2016). Montmorillonite functions as a bioinspired building block to target bacteria, while PEI can cause bacterial membrane lysis, allowing increased antibiotic entrance into cytoplasm. The orally applied nanoparticles were observed to largely distribute in gastric tissue, confirming their mucoadhesive capability. The nanocarriers were able to eradicate *H. pylori* in GI for improving gastric ulcer and the inflammation response. This antimicrobial efficiency was greater than that of the conventional triple therapy (omeprazole/amoxicillin/metronidazole). For targeting to the infection site of *H. pylori*, gastric epithelial cell membrane was coated onto PLGA nanoparticles for preparing biomimetic nanocarriers containing clarithromycin as the model antibiotic (Angsantikul et al., 2018). The affinity of *H. pylori* with the biomimetic nanocarriers was 10-fold higher than that with non-coated nanoparticles. The bacterial burden in stomach of the infected mice (1.6 × 10^5^ CFU/g) was reduced to 6.5 × 10^3^ and 5.0 × 10^4^ CFU/g by oral delivery of the biomimetic nanoparticles and free drug, respectively.

### The Other Infection Sites

The antibacterial nanoparticles were also active to treat systemic, bone, and vaginal infections. The systemic bacterial infection can prompt bacteremia, followed by the occurrence of sepsis (Huttunen and Aittoniemi, 2011). Rai et al. (2016) conjugated antimicrobial peptide on the Ag nanoparticle surface with high density to eradicate MRSA strain in circulation. The model peptide employed in this study was cecropin-melittin. The nanoparticle size could be controlled to 14 nm. The Au nanoparticles were intraperitoneally injected into the sepsis-like mice to treat bacteremia. The peptide-conjugated nanoparticles decreased MRSA concentration by 2 logs in the circulation compared to the non-conjugated group. Most of the nanoparticles would be distributed to the spleen. Metallic nanoparticles were also utilized to treat bone infection.

Qadri et al. (2017) developed Ag-Cu nanoparticles to eradicate *S. aureus* bone invasion in mice. Boron was added in the nanoparticles to prolong antimicrobial activity since the anticorrosive feature of boron was valuable in delaying Cu oxidation (Prasai et al., 2012). The nanoparticulate diameter was about 27 nm. *S. aureus* was incorporated in silk suture and then implanted into the bone of mice to induce osteomyelitis. The intravenous nanoparticle administration at 1 mg/kg reduced bacterial CFU by 10-fold compared to the control. The intramuscular route showed a similar tendency to suppress *S. aureus* accumulation in bone. Osteomyelitis was also treated by magnetic Fe_3_O_4_ nanoparticles with the induction of hyperthermia to disrupt biofilm (Fang et al., 2017). The SPIONs were implanted in bone infected by *S. aureus*. The inserted implant could be heated to 75°C by magnetic field targeting in the infected bone. The local administration of vancomycin into the femoral canal in the presence of hyperthermia promoted the bacterial eradication in the biofilm. The bone volume of the combined vancomycin and hyperthermia (24%) was greater than that of the infection control (18%). ZnO nanoparticles were proved to display antimicrobial activity at very low concentration (Król et al., 2017). ZnO nanoparticles with the size of 10 nm were incorporated in PVA hydrogel for treating vaginitis via vaginal administration (Bai et al., 2015). The vaginitis mice were established by inoculating *E. coli* in vagina of mice for 5 d. The CFU in vaginal washes was significantly cleared after treatment of the nanoparticles. *E. coli* counting and epithelial exfoliation score determined by histology showed a consistent tendency.

## Conclusion

When considering the formulation design for antibacterial therapy, it is important to develop the carriers that can stabilize the active ingredients and improve delivery into the infection sites. Increasing attention has been paid to nanomedicine as an approach for applying bacterial targeting or delivery. The use of antibiotic-loaded nanoparticles is considered a valid strategy for bacteria targeting because of its numerous advantages over conventional formulations, including improved stability, controlled antibiotic release, targeted capability, and increased bioavailability. Some issues, such as solubility, drug resistance, and epithelium permeation, can also be resolved by nanocarriers' targeting pathogens. Though many nanocarriers have been developed for testing in cell-based and animal studies, clinical trials for bacteria-delivery application are still limited. This may be due to the high cost of clinical trials and the unknown side effects that should be first identified and explored. Nanosystems are thought to cause more serious adverse effects on organisms compared to the bulk materials, as their very small size causes a correspondingly higher surface area. The researchers should pay attention only not to nanoparticles' therapeutic benefits but also to their toxic responses on human health. Caution should be used in optimizing the feasible conditions of nanomedicine for balancing the effectiveness of antimicrobial therapy and tissue damage. For use in future human applications, the materials utilized for preparing antibacterial nanoparticles should be non-toxic, biodegradable and biocompatible. The materials approved by the FDA may be the first choices for the development of these nanocarriers. There are already some experiences of antibiotic-loaded nanoparticles approved by FDA for clinical application. It is expected that more antibacterial nanoparticles will be released for clinical use. Because of the improvement of industrial preparation and scale-up in the recent years, the visibility of commercially antibacterial nanoparticles can be raised in the near future. The introduction and description of the nanocarriers for bacterial and infection site targeting outlined in this review may provide relevant information to investigators involved in designing feasible and efficient delivery systems for the treatment of bacteria and its related diseases. It is preferable if some suggestions can be made for selecting the nanoparticles with best antibacterial efficiency. However, this intention is difficult to achieve since different investigations involved in the development of antibacterial nanoparticles employ different evaluation platforms. Although the MIC is the assay most-frequently used, the protocols of MIC determination are always different among different studies. Thus it should be cautious to compare the antimicrobial activity of the various types of nanosystem. Nevertheless, the comparison of antibacterial effect of the nanoparticles with a positive control antibiotic approved for clinical application is suggested.

## Author Contributions

J-YF conceived the topic of the review article and wrote most of the draft. Y-CY and T-HH took part in the writing and the discussion. S-CY and C-CC wrote sections of the review and prepared the figures. All the authors made comments and suggestions for the writing of the review.

## Conflict of Interest

The authors declare that the research was conducted in the absence of any commercial or financial relationships that could be construed as a potential conflict of interest.
